# Inhibition of USP1 reverses the chemotherapy resistance through destabilization of MAX in the relapsed/refractory B-cell lymphoma

**DOI:** 10.1038/s41375-022-01747-2

**Published:** 2022-11-09

**Authors:** Xi-Ya Li, Ji-Chuan Wu, Ping Liu, Zi-Juan Li, Yong Wang, Bing-Yi Chen, Cheng-Long Hu, Ming-Yue Fei, Peng-Cheng Yu, Yi-Lun Jiang, Chun-Hui Xu, Bin-He Chang, Xin-Chi Chen, Li-Juan Zong, Jia-Ying Zhang, Ying Fang, Xiao-Jian Sun, Kai Xue, Li Wang, Shu-Bei Chen, Shi-Yu Jiang, Ai-ling Gui, Ling Yang, Juan J. Gu, Bao-Hua Yu, Qun-ling Zhang, Lan Wang

**Affiliations:** 1 CAS Key Laboratory of Tissue Microenvironment and Tumor, Shanghai Institute of Nutrition and Health, University of Chinese Academy of Sciences, Shanghai Jiao Tong University School of Medicine (SJTUSM) & Chinese Academy of Sciences, Shanghai, China; 2 Shanghai Institute of Hematology, State Key Laboratory of Medical Genomics, National Research Center for Translational Medicine at Shanghai, Ruijin Hospital Affiliated to Shanghai Jiao Tong University School of Medicine, Shanghai, China; 3 Shanghai Jiao Tong University School of Life Sciences and Biotechnology, Shanghai, China; 4 Department of lymphoma, Fudan University Shanghai Cancer Center, Shanghai, China; 5 Department of Cellular and Genetic Medicine, School of Basic Medical Sciences, Fudan University, Shanghai, China; 6 Department of Medicine & Immunology, Roswell Park Cancer Institute, Buffalo, NY USA; 7 Department of Pathology, Fudan University Shanghai Cancer Center, Shanghai, China

**Keywords:** B-cell lymphoma, Haematological diseases

## Abstract

The patients with relapsed and refractory diffuse large B-cell lymphoma (DLBCL) have poor prognosis, and a novel and effective therapeutic strategy for these patients is urgently needed. Although ubiquitin-specific protease 1 (USP1) plays a key role in cancer, the carcinogenic effect of USP1 in B-cell lymphoma remains elusive. Here we found that USP1 is highly expressed in DLBCL patients, and high expression of USP1 predicts poor prognosis. Knocking down USP1 or a specific inhibitor of USP1, pimozide, induced cell growth inhibition, cell cycle arrest and autophagy in DLBCL cells. Targeting USP1 by shRNA or pimozide significantly reduced tumor burden of a mouse model established with engraftment of rituximab/chemotherapy resistant DLBCL cells. Pimozide significantly retarded the growth of lymphoma in a DLBCL patient-derived xenograft (PDX) model. USP1 directly interacted with MAX, a MYC binding protein, and maintained the stability of MAX through deubiquitination, which promoted the transcription of MYC target genes. Moreover, pimozide showed a synergetic effect with etoposide, a chemotherapy drug, in cell and mouse models of rituximab/chemotherapy resistant DLBCL. Our study highlights the critical role of USP1 in the rituximab/chemotherapy resistance of DLBCL through deubiquitylating MAX, and provides a novel therapeutic strategy for rituximab/chemotherapy resistant DLBCL.

## Introduction

Lymphoma is a type of malignant tumor originating from lymphocytes and is divided into Hodgkin’s lymphoma (HL) and non-Hodgkin’s lymphoma (NHL). Diffuse large B-cell lymphoma (DLBCL) is the most common subtype of lymphoma, accounting for 30-35% of NHL [1, 2]. In recent years, 6–8 cycles of R-CHOP (rituximab, cyclophosphamide, doxorubicin, vincristine and prednisone) have become the standard treatment for DLBCL [3–7], and a good efficacy of the R-CHOP regimen has been achieved. However, some DLBCL patients with poor prognosis features are still resistant to therapy or relapse after short-term remission. Thus, it is very important to identify markers associated with the prognosis of DLBCL patients and therapeutic efficacy of R-CHOP regimen, which will ultimately help explore new targeted therapy of rituximab/chemotherapy resistant DLBCL.

USP1, a deubiquitinating enzyme, is a member of the ubiquitin-specific processing (USP) family of proteases [8]. USP1 has conserved USP-domain and Cys-box/His-box motifs, which contain catalytic residues (Cys90, His593 and Asp751) [8–10]. USP1 is located in both cytoplasm and nucleus and cleaves the ubiquitin moiety from ubiquitinylated proteins. Ectopic expression of USP1 in mesenchymal stem cells inhibited osteoblastic differentiation, and enhanced cell proliferation through stabilizing ID (inhibitor of DNA binding) proteins [11].

USP1 has been also implicated in cancer progression [12]. USP1 can deubiquitinate ID1 (inhibitor of DNA binding 1), a transcription regulator, which was identified to control leukemogenesis by us previously, and protected ID1 from proteasome-mediated degradation [13]. Accordingly, USP1 could be a therapeutic target for the treatment of cancer. Pimozide, a specific and reversible inhibitor of the USP1/UAF1 deubiquitinase complex, was obtained through the high-throughput screening [14]. It was reported that pimozide inhibited the viability of acute myeloid leukemia (AML) cells by promoting the degradation of the USP1 substrate ID1 [13]. We previously found that pimozide prolonged the survival time of t(8;21) AML mice [15]. Therefore, a better understanding of how USP1 regulates carcinogenesis could improve the prevention and treatment of cancer. So far, the biological function and underlying mechanism of USP1 in DLBCL have been undefined.

Here we investigated the function and pathogenic mechanism of USP1 in DLBCL. Our clinical analysis showed that USP1 is highly expressed in DLBCL patients, which predicts poor prognosis. Furthermore, the abrogation of USP1 expression by shRNA knockdown induced apoptosis and autophagy in DLBCL cells and impaired the tumorigenic activity of these cells, which caused the reversion of chemotherapy resistance. Inhibition of USP1 in DLBCL cells led to decreased expression of MAX and MYC and subsequently suppressed the activation of MYC and its downstream targets. Moreover, pimozide, the USP1 inhibitor, reversed the chemotherapy resistance in the relapsed/refractory DLBCL. Overall, this study highlights that targeting USP1 may provide an effective therapeutic strategy for the treatment of DLBCL.

## Materials and methods

### Patients and samples

A total of 106 newly diagnosed DLBCL patients were included in this retrospective study. The diagnosis of DLBCL was reviewed by experienced pathologists in the pathology department of Fudan University Shanghai Cancer Center, Shanghai, China. All patients were under standard treatment with R-CHOP-based regimen at the Fudan University Shanghai Cancer Center from April 2009 to December 2018. This study was approved by the Institutional Review Board of Fudan University Shanghai Cancer Center. All patients signed informed consent forms to review their medical records and research. Survival data were available with a median follow-up of 78.6 months (range from 2.7 to 117.6 months). The normal lymph nodes were included in a tissue microarray (Cat. No. ILY-2086a, Alenabio). Primary lymphoma cells #1 were obtained by lymph node puncture from the node of a 57-year-old male DLBCL patient who relapsed after 6 cycles of R-CHOP. Primary lymphoma cells #2 were obtained from the tonsil biopsy of a 53-year-old female patient with DLBCL who relapsed after 6 cycles of R-CHOP. Primary lymphoma cells #3 were obtained from the node of a 55-year-old female patient, diagnosed with stage IV DLBCL, who relapsed after 6 cycles of R-CHOP, confirmed by breast lesion biopsy.

### Cell lines

DLBCL cell lines (RL, RL-4RH, U2932, SUDHL4, TDM8, RIVA and DB cells) were used. The RL [germinal center B-cell (GCB) DLBCL] and RL-4RH (rituximab/chemotherapy resistant B-cell lymphoma cell line, RRCL) were kind gifts given by Czuczman. All cells were generated and characterized from rituximab/chemotherapy sensitive B-cell lymphoma cell line (RSCL) as previously described [16]. All cell lines were cultured at 37 °C in RPMI 1640 with Glutamax-1 (Gibco, C11875500CP) supplemented with 10% heat-inactivated fetal bovine serum, HEPES (5 mM), penicillin and streptomycin (100 IU/mL) and sodium pyruvate (1 mM).

### MTT assay

Cells were plated at a density of 1 × 10^5^ cells/mL in 96-well plates (50 µL/well) and exposed to different concentrations of pimozide and etoposide, either alone or in combinations. 0.1 mg MTT was added to each well. After the incubation at 37 °C for 4 h, formazan was decomposed by 50 µL triple lysis buffer (10% SDS, 5% isopropanol and 0.012 M HCL) overnight and then the absorbance was measured at 562 nm by spectrophotometry.

## Results

### Demographic and baseline characteristics of patients

106 newly diagnosed DLBCL patients treated with R-CHOP were enrolled in our study, and all the patients had an Eastern Cooperative Oncology Group (ECOG) performance of 0–1. The median age was 50 years (range: 19-73 years) at the time of diagnosis. 20 patients (18.9%) were more than 60 years old. 67 patients (63.2%) had stage I/II diseases, and 39 patients (36.8%) had stage III/IV diseases according to Ann Arbor classification. There were 18 patients (17.0%) with more than one extranodal lesion in this study. 34 patients (32.1%) had elevated lactate dehydrogenase (LDH). 26 patients (24.5%) had bulky disease and 20 patients (18.9%) had B symptoms. There were 45 patients (42.5%) with germinal center B-cell lymphoma (GCB), 45 (42.5%) patients with non-germinal center B-cell lymphoma (non-GCB) and 16 patients (15.1%) with unknown cell of origin in this study. It is very interesting that USP1 was highly expressed in female patients with DLBCL (*P* = 0.048). USP1 expression in the 106 DLBCL patients treated with R-CHOP was significantly associated with the risk of bulky disease (*P* = 0.005), and USP1 high-expression group exhibited elevated LDH (Table 1). Multivariate analysis of USP1 associated with PFS and OS showed that USP1 was significantly associated with PFS (Supplementary Fig. S1a). To further study the crosstalk mechanistically between USP1 and those newly established genetic subtypes that carry a poor outcome, we collected the additional 59 cases of relapsed/refractory (newly diagnosed) DLBCL patient samples containing MCD (including MYD88^L265P^ and CD79B mutations), N1 (including NOTCH1 mutations) subtypes and analyzed the expression of USP1 by using immunohistochemistry analysis. The results showed that the positive rates of USP1 in patients with MCD and N1 subtypes were 63% and 33%, and USP1 was highly expressed in MCD subtype patients (Supplementary Fig. S1b).

**Table 1:** USP1 expression in 106 DLBCL patients and their baseline characteristics.

		USP1 expression		*p* value
	*n*	Negative (*n*,%)	Positive (*n*,%)	
Gender				
Female	51	15 (29.4)	36 (70.6)	0.048
Male	55	27 (49.1)	28 (50.9)	
Age, years				
Median (range)		47(24-68)	50.5(19-73)	
≦60	86	35 (40.7)	51 (59.3)	0.801
>60	20	7 (35.0)	13 (65.0)	
Performance status				
0	68	28 (41.2)	40 (58.8)	0.685
1	38	14 (36.8)	24 (63.2)	
Ann Arbor Stage				
I–II	67	27 (40.3)	40 (59.7)	1
III–IV	39	15 (38.5)	24 (61.5)	
Number of extranodal sites				
0–1	88	39 (44.3)	49 (55.7)	0.035
>1	18	3 (16.7)	15 (83.3)	
LDH level				
≦Normal	72	33 (45.8)	39 (54.2)	0.088
>Normal	34	9 (26.5)	25 (73.5)	
IPI score				
0	50	22 (44.0)	28 (56.0)	0.197
1	21	7 (33.3)	14 (66.7)	
2	21	11 (52.4)	10 (47.6)	
3	13	2 (15.4)	11 (84.6)	
4	1	0 (0)	1 (100)	
B symptom				
Yes	20	6 (30.0)	14 (70.0)	0.448
No	86	36 (47.5)	50 (58.1)	
Bulky disease				
Yes	26	4 (15.4)	22 (84.6)	0.005
No	80	38 (47.5)	42 (52.5)	
Cell of Origen				
GCB	45	17 (37.8)	28 (62.2)	0.329
Non-GCB	45	16 (35.6)	29 (64.4)	
Unknown	16	9 (56.3)	7 (43.7)	

*GCB* germinal center B cell-like, *LDH* lactate dehydrogenase, *IPI* International Prognostic Index.

*p < 0.05.

### USP1 is highly expressed in DLBCL and high expression of USP1 is associated with poor prognosis

**Fig. 1:**
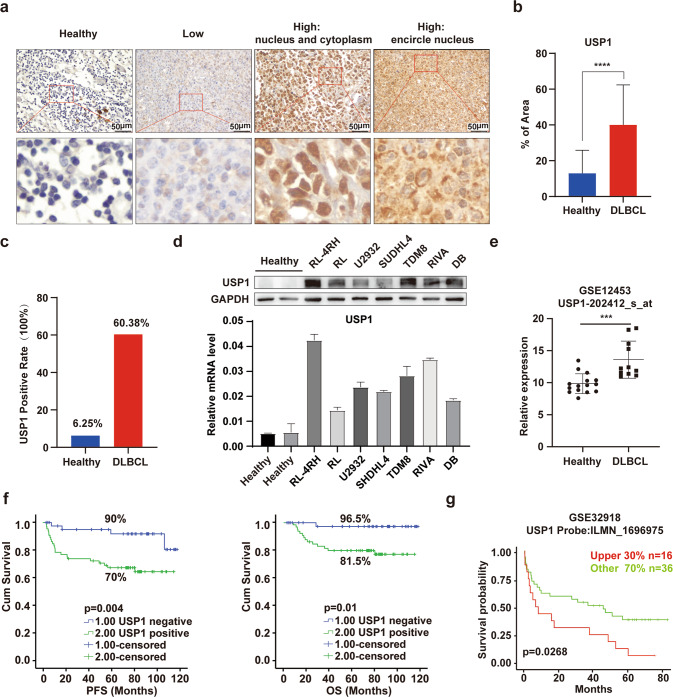
USP1 is highly expressed in DLBCL and associated with poor prognosis. **a** Immunohistochemical staining analysis for USP1 in primary DLBCL samples and healthy subject samples [Healthy: healthy subject samples, Low: low expression of USP1, High (nucleus and cytoplasm): high expression of USP1 in nucleus and cytoplasm, High (encircle nucleus): high expression of USP1 in the encircle nucleus]. Scale bar = 50 μm. **b** Quantitative results of immunohistochemical assays for USP1 in primary DLBCL samples. **c** The USP1-positive rates of DLBCL patients or healthy subjects were calculated (negative: lower than 55%, positive: greater than or equal to 55%). **d** Western blotting and quantitative real-time PCR analysis of USP1 expression in DLBCL cell lines and healthy human PBMCs. **e** The relative ratio of USP1 mRNA in DLBCL tissue samples versus that in normal B cells was shown. Data were obtained from the GEO database. **f** Conjoint analysis of USP1 expression and the prognosis of DLBCL patients. **g** The analysis of GSE32918 data by using LOGpc indicated that patients with higher USP1 expression had shorter OS than those with lower USP1 expression. Data are presented as mean ± SD, *p < 0.05, **p < 0.01, ***p < 0.005, ****p < 0.001.

To elucidate the potential role of USP1 in DLBCL, we detected the expression of USP1 by using immunohistochemical (IHC) analysis in 106 newly diagnosed DLBCL samples and 16 normal lymph node tissue samples. We found that USP1 was expressed in the nucleus and cytoplasm (Fig. 1a, b). Tumors were considered USP1-positive if USP1 was expressed in the nucleus of >55% of tumor cells. Our result showed that the USP1 expression levels of DLBCL samples was strikingly higher (64/106) than that of normal lymph nodes (1/16) (Fig. 1c). In addition, the USP1 expression level of human DLBCL cell lines was significantly higher than that of normal peripheral blood mononuclear cells (PBMC) (Fig. 1d). To explore the transcriptional alterations of USP1 between DLBCL samples and normal B cells, we analyzed the GSE database [17], and found that USP1 expression was markedly elevated in DLBCL patients compared with healthy subjects (Fig. 1e).

Subsequently, we analyzed the correlation between the USP1 expression level and the prognosis of DLBCL patients and found that high USP1 expression was associated with a worse outcome. The 5-year progression-free survival (PFS) in the USP1-positive group was shorter than that in the USP1-negative group (70.0% *vs* 90.0%, *p* = 0.004). Moreover, the 5-year overall survival (OS) in the USP1-positive group was shorter than that in the USP1-negative group (81.5% *vs* 96.5%, *p* = 0.01) (Fig. 1f). We found that the DLBCL patients with high expression of USP1 had significantly shorter OS by using LOGpc analysis, an online analysis tool developed by biomedical informatics institute (Fig. 1g). These results indicated that overexpression of USP1 in DLBCL was associated with an unfavorable prognosis.

### Knockdown of USP1 inhibited the proliferation of rituximab/chemotherapy resistant DLBCL cells in vitro and in vivo

**Fig. 2:**
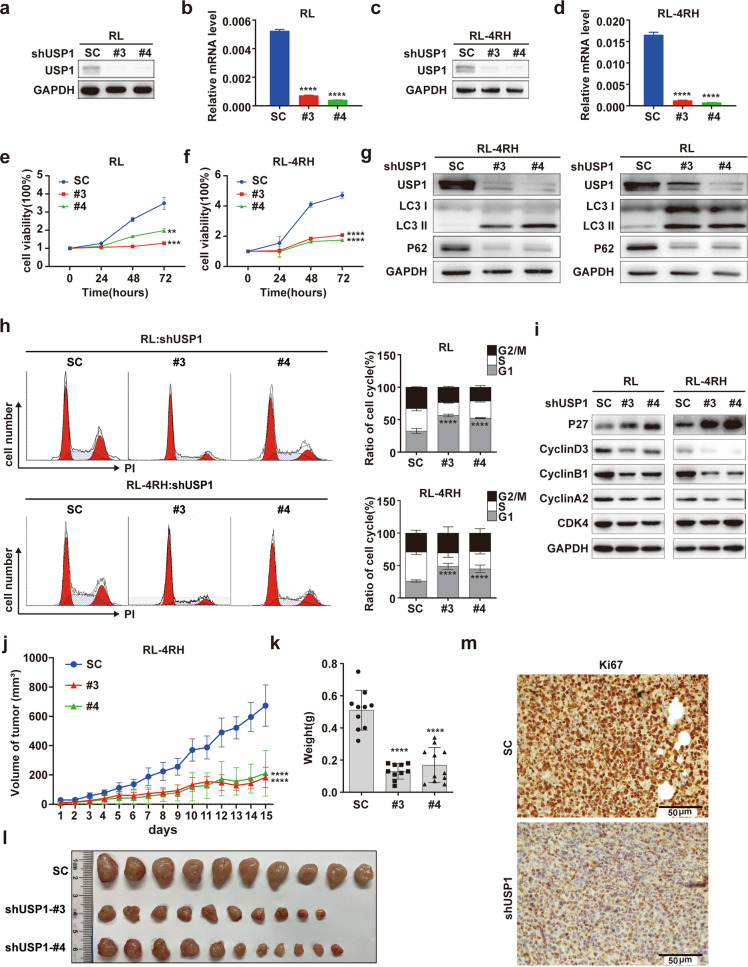
Knockdown of USP1 inhibited the proliferation of rituximab/chemotherapy resistant DLBCL cells in vitro and in vivo. **a**–**d** Western blotting and quantitative real-time PCR analysis of USP1 expression in RL and RL-4RH cells with USP1 knockdown and the control cells. **e**, **f** The proliferation of RL and RL-4RH cells transduced with shRNA against USP1 (shUSP1) or the scrambled shRNA (shSC) was examined by using MTT assay. **g** Autophagy-related proteins were examined by using western blotting assay in RL and RL-4RH cells with USP1 knockdown. **h** Changes in the cell cycle were examined by using flow cytometry assays in RL and RL-4RH cells with USP1 knockdown. The representative pictures of flow cytometry analysis (left panel) and the statistical results of flow cytometry experiments (right panel) were shown. **i** Changes in cell cycle-related proteins were examined by using western blotting assay in RL and RL-4RH cells with USP1 knockdown. **j**–**l** A DLBCL xenograft mouse model was established by using RL-4RH cells with or without USP1 knockdown. **m** The expression levels of Ki67 in xenograft tumors were determined by using IHC assay. Data are presented as mean ± SD from three independent experiments, *p < 0.05, **p < 0.01, ***p < 0.005, ****p < 0.001. Statistical analysis was performed with a paired *t* test.

To further study the role of USP1 in DLBCL, we used two shRNAs against USP1, which remarkably reduced the mRNA and protein levels of USP1 in RL-4RH (rituximab/chemotherapy resistant DLBCL cells), RL, U2932 and SUDHL4 cells (Fig. 2a–d and Supplementary Fig. S2a). To examine the effect of USP1 on the proliferation of DLBCL cells, we performed MTT assay, and found that knocking down of USP1 significantly suppressed the proliferation of DLBCL cells (Fig. 2e, f and Supplementary Fig. S2b). To determine whether the wild-type USP1 or catalytically inactive mutant of USP1 can rescue the phenotype of USP1 knockdown in DLBCL cells, we first knocked down USP1 and then overexpressed wild-type USP1/catalytically inactive mutant of USP1 in RL-4RH cells. The results showed that overexpression of wild-type USP1, but not catalytically inactive mutant of USP1, restored the proliferation of RL-4RH cells with USP1 knockdown (Supplementary Fig. S2c). Flow cytometry analysis and Wright’s staining were performed to evaluate the apoptosis of RL cells. The results demonstrated that knocking down USP1 induced apoptosis in RL cells, but not in RL-4RH cells (Supplementary Fig. S2d, e), which is consistent with the previous report that RL-4RH cells were unable to undergo apoptosis due to loss of BAX/BAK expression [18]. To explore the mechanism of RL-4RH cell death induced by inhibition of USP1, we evaluated the protein levels of p62 and LC3, the autophagy markers. We found that knockdown of USP1 increased LC3 protein levels and decreased p62 protein levels in RL-4RH cells (Fig. 2g), which indicated that the autophagic activity was increased by inhibition of USP1. In addition, knocking down USP1 induced cell cycle arrest at G0/G1 phase in RL, RL-4RH, U2932 and SUDHL4 cells (Fig. 2h and Supplementary Fig. S2f). To examine the change of cell cycle-related proteins, we performed western blotting analysis in RL and RL-4RH cells with USP1 knockdown. The results showed that knockdown of USP1 induced the upregulation of cell cycle-arresting protein such as P27 and downregulation of proteins promoting cell cycle progression such as CyclinB1, CyclinA2, CyclinD3 and CDK4 (Fig. 2i). Therefore, silencing USP1 inhibited the growth of DLBCL cells, induced autophagy and cell cycle arrest in RL and RL-4RH cells.

To explore the effect of USP1 on DLBCL cells in vivo, we established a DLBCL xenograft mouse model using RL-4RH cells. We found that tumors with knockdown of USP1 displayed significantly reduced growth rates compared with control tumors (Fig. 2j). The tumor weight of the mice in USP1 knockdown group was significantly lower than that of the control mice (Fig. 2k, l). The decreased levels of Ki67, the cell proliferation-related protein, was observed in tumors with knockdown of USP1 (Fig. 2m). Collectively, these results demonstrated that the knockdown of USP1 in vivo inhibited the proliferation of rituximab/chemotherapy resistant DLBCL cells.

### USP1 interacted with MAX and regulated the stability of MAX through deubiquitination in rituximab/chemotherapy resistant DLBCL cells

**Fig. 3:**
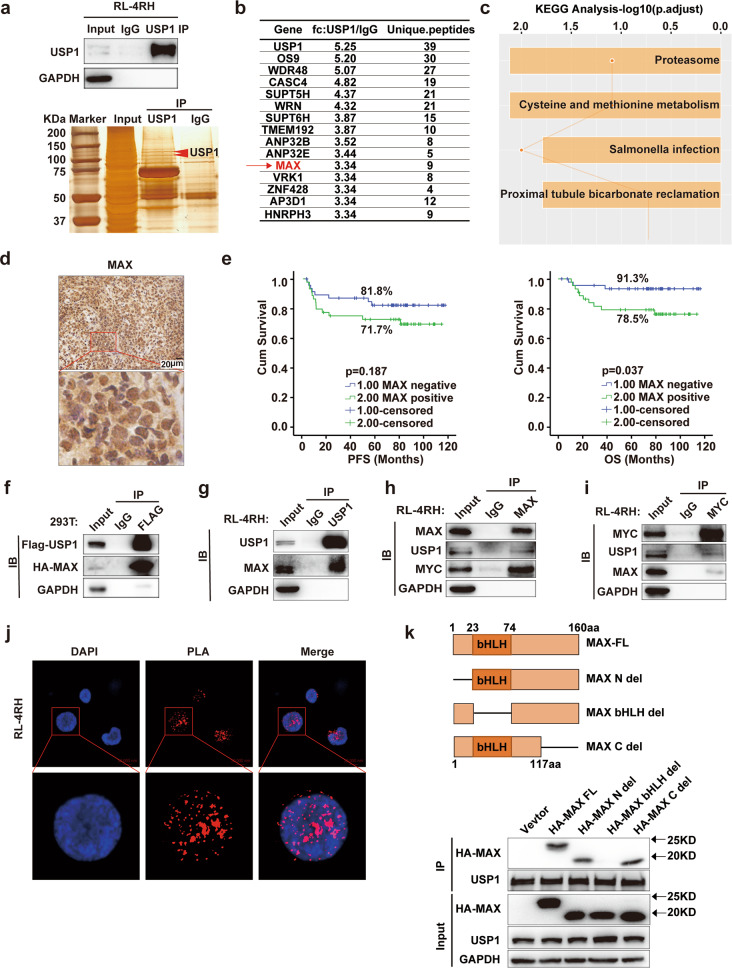
USP1 interacted with MAX and MYC in rituximab/chemotherapy resistant DLBCL cells. **a** The IP assay was performed by using an anti-USP1 antibody in RL-4RH cells, and the enriched proteins were examined by using western blotting and silver nitrate staining assays. **b** The list of the top 15 USP1-interacting proteins. **c** KEGG pathway analysis of USP1-interacting proteins. **d** Immunohistochemical staining analysis for MAX in primary DLBCL samples. **e** Conjoint analysis of MAX expression and the prognosis of DLBCL patients. **f** 293T cells were transfected with Flag-USP1 and HA-MAX. USP1 was immunoprecipitated with an anti-Flag antibody and HA-MAX was examined by using western blotting assay. The control immunoprecipitation was performed with IgG. **g** USP1 was immunoprecipitated with anti-USP1 antibody in RL-4RH cells and MAX was examined by using western blotting assay. The control immunoprecipitation was performed by using IgG. **h**, **i** MAX and MYC were immunoprecipitated in RL-4RH cells with anti-MAX or anti-MYC antibody, and USP1 was detected by using western blotting assay. **j** Proximity ligation assay (PLA) assay to detect the interaction of USP1 and MAX. **k** Co-immunoprecipitation and western blotting analysis showing the binding of HA-MAX and its variants with USP1 in 293T cells. Data are presented as mean ± SD, *p < 0.05, **p < 0.01, ***p < 0.005, ****p < 0.001.

To further elucidate the molecular mechanisms underlying the pro-proliferative effects of USP1 in DLBCL, we characterized the USP1 interactome by using mass spectrometry analysis. The whole cell lysates of RL-4RH cells were subjected to immunoprecipitation using anti-USP1 antibody, and the result of silver nitrate staining showed that USP1 was immunoprecipitated from the cell lysates (Fig. 3a). We identified the top 15 proteins interacting with USP1 by using the mass spectrum analysis (Fig. 3b). KEGG analysis revealed that the proteins interacting with USP1 were significantly enriched in the ubiquitin-proteasome pathway (Fig. 3c). To find the potential targets of USP1 in DLBCL, we first analyzed the proteins interacting with USP1 in the GEO database, and the results showed that the expression levels of MAX in DLBCL patient samples were significantly higher than those of healthy controls (Supplementary Fig. S3a). Subsequently, we detected the expression of MAX by using immunohistochemical (IHC) analysis in newly diagnosed DLBCL samples. Tumors were considered MAX-positive if MAX was expressed in the nucleus of >30% of tumor cells. Next, we analyzed the correlation between the MAX expression level and the prognosis of DLBCL patients and found that high MAX expression was associated with a worse outcome. The 5-year progression-free survival (PFS) in the MAX-positive group was shorter than that in the MAX-negative group (71.7% *vs* 81.8%, *p* = 0.187). The 5-year overall survival (OS) in the MAX-positive group was shorter than that in the MAX-negative group (78.5% *vs* 93.1%, *p* = 0.037) (Fig. 3d, e). Multivariate analysis of MAX associated with PFS and OS showed that MAX was not an independent prognostic factor (Supplementary Fig. S3b). We then analyzed the expression of MAX in a sample of 59 newly diagnosed relapsed/refractory DLBCL patients. The results showed that the positive rates of MAX in patients with MCD and N1 subtypes were 75% and 33%, and MAX was highly expressed in MCD subtype patients (Supplementary Fig. S3c, d). These results suggested that MAX may be a potential target of USP1 in DLBCL cells.

**Fig. 4:**
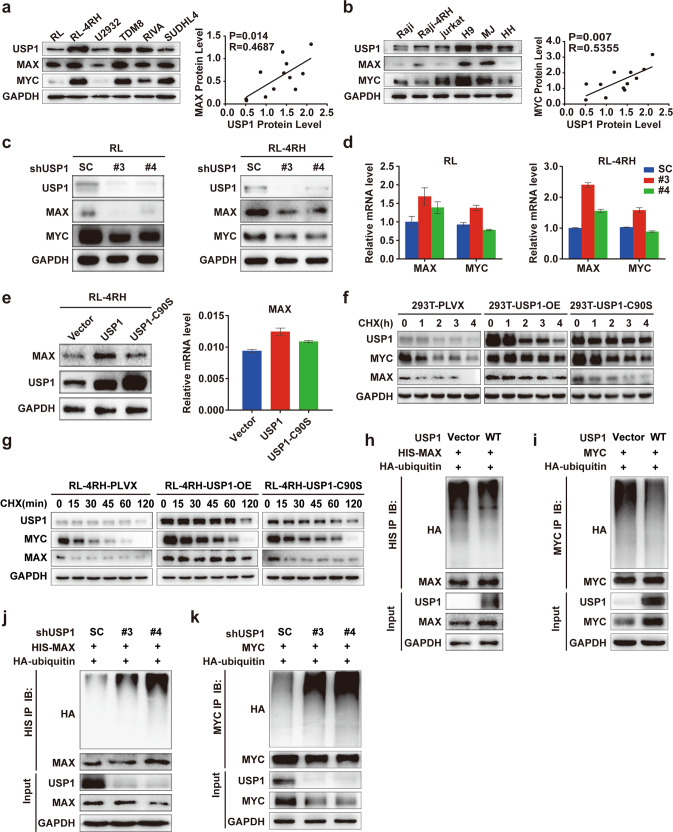
USP1 maintained the stability of MAX through deubiquitination in rituximab/chemotherapy resistant DLBCL cells. **a**, **b** The expression of USP1, MAX and MYC was examined by using western blotting assay in DLBCL, Burkitt and T cell lymphoma cell lines. **c**, **d** Western blotting and quantitative real-time PCR analysis of USP1, MAX and MYC expression were performed in RL and RL-4RH cells with USP1 knockdown. **e** Western blotting and quantitative real-time PCR analysis of MAX in RL-4RH cells overexpressing USP1, USP1-C90S, or the empty vector. **f** 293T cells, transduced with USP1, USP1-C90S or the empty vector, were treated with CHX for 1, 2, 3 or 4 h and the expression of USP1, MAX and MYC was examined by using western blotting assay. **g** RL-4RH cells, transduced with USP1, USP1-C90S or the empty vector, were treated with CHX for 15, 30, 45, 60 or 120 min and the expression of USP1, MAX and MYC was examined by using western blotting assay. **h** 293T cells overexpressing MAX were transfected with USP1, HA-ubiquitin or the empty vector. MAX was immunoprecipitated with anti-HIS antibody, and HA-ubiquitin was examined by using western blotting assay. **i** 293T cells overexpressing MYC were transfected with USP1, HA-ubiquitin or the empty vector. MYC was immunoprecipitated with anti-MYC antibody, and HA-ubiquitin was examined by using western blotting assay. **j** 293T cells overexpressing MAX were transfected with HA-ubiquitin, and transduced with shRNA (#3, #4) against USP1 or the control shRNA (SC) 24 h after transfection. MAX was immunoprecipitated with anti-HIS antibody, and HA-ubiquitin was examined by using western blotting assay. **k** 293T overexpressing MYC were transfected with HA-ubiquitin and then transduced with shRNA against USP1 or the control shRNA 24 h after transfection. MYC was immunoprecipitated with anti-MYC antibody, and HA-ubiquitin was examined by using western blotting assay.

MAX has been shown to form a heterodimer with MYC and plays an important role in lymphomagenesis [19, 20]. To explore whether MAX is the target of USP1 in DLBCL, we first validated the interaction between USP1 and MAX. We transfected HA-MAX and Flag-USP1 into 293T cells and observed an interaction between USP1 and MAX in these cells by co-immunoprecipitation (Co-IP) assays (Fig. 3f). The interactions of endogenous USP1 and MAX/MYC were further confirmed by Co-IP assays in RL-4RH cells (Fig. 3g–i). Proximity ligation assay (PLA) demonstrated that USP1 strongly interacted with MAX in RL-4RH cells (Fig. 3j). To determine the domains of MAX that are used to interact with USP1. We overexpressed MAX WT and MAX truncates in 293T cells, and then co-immunoprecipitation (Co-IP) assays was performed with USP1 antibody. The results showed USP1 interacts with the bHLH domain of MAX (Fig. 3k). Moreover, the results of GST pull-down assay showed that USP1 directly bound to MAX/MYC (Supplementary Fig. S3e, f). Then, we assessed whether USP1 could regulate MAX expression in DLBCL cells. We first analyzed the correlation of USP1 and MAX expression in DLBCL patient samples, and the results showed that the expression of USP1 is positively correlated with the expression of MAX (Supplementary Fig. S3g, h). Subsequently, we detected the expression of USP1, MAX and MYC in DLBCL, Burkitt and T cell lymphoma cell lines by using western blotting analysis, and found that the protein levels of USP1, MAX and MYC were high in RL-4RH cells (Fig. 4a, b). Knocking down USP1 significantly decreased the protein levels of MAX and MYC in RL and RL-4RH cells (Fig. 4c) and overexpression of USP1 markedly increased the protein levels of MAX in RL-4RH cells (Fig. 4e). Knocking down USP1 significantly decreased the expression of MYC target genes (CyclinB1, CyclinA2 and E2F2) in RL-4RH cells (Supplementary Fig. S4a). Interestingly, neither knockdown nor overexpression of USP1 affected the mRNA levels of MAX and MYC in RL and RL-4RH cells (Fig. 4d, e). Therefore, we further determined whether the interaction between USP1 and MAX is functionally required for the upregulation of MAX. We found that overexpression of USP1 increased the stability of MAX and MYC protein in 293T and RL-4RH cells treated with cycloheximide (CHX) (Fig. 4f, g). These results indicated that USP1 was involved in post-translational regulation of MAX and MYC in DLBCL cells. To validate these findings, we performed ubiquitylation assays and examined whether USP1 regulated the ubiquitination of MAX and MYC. USP1 and HA-ubiquitin plasmids were co-transfected into 293T cells overexpressing MAX or MYC. Then, we performed the immunoprecipitation assay by using anti-HIS or anti-MYC antibodies in these cells and observed a significant reduction in the ubiquitination of MAX and MYC proteins (Fig. 4h, i). Accordingly, the ubiquitination levels of MAX and MYC were significantly increased in 293T cells with USP1 knockdown (Fig. 4j, k). To determine which the lysine residues in MAX deubiquitinated by USP1, we mutated K24, K40 or K57, which were predicted as potential ubiquitylation sites of MAX [21]. The results showed that only the K57R mutation caused significant attenuation of MAX ubiquitylation (Supplementary Fig. S4b). Furthermore, USP1 knockdown enhanced ubiquitylation of the MAX^WT^ but not MAX^K57R^ mutant (Supplementary Fig. S4c). Together, these data demonstrated that USP1 deubiquitinated and stabilized MAX and MYC proteins in rituximab/chemotherapy resistant DLBCL cells.

### Overexpression of MAX or MYC rescued the inhibition of cell proliferation induced by USP1 knockdown in cell and mouse models of rituximab/chemotherapy resistant DLBCL

**Fig. 5:**
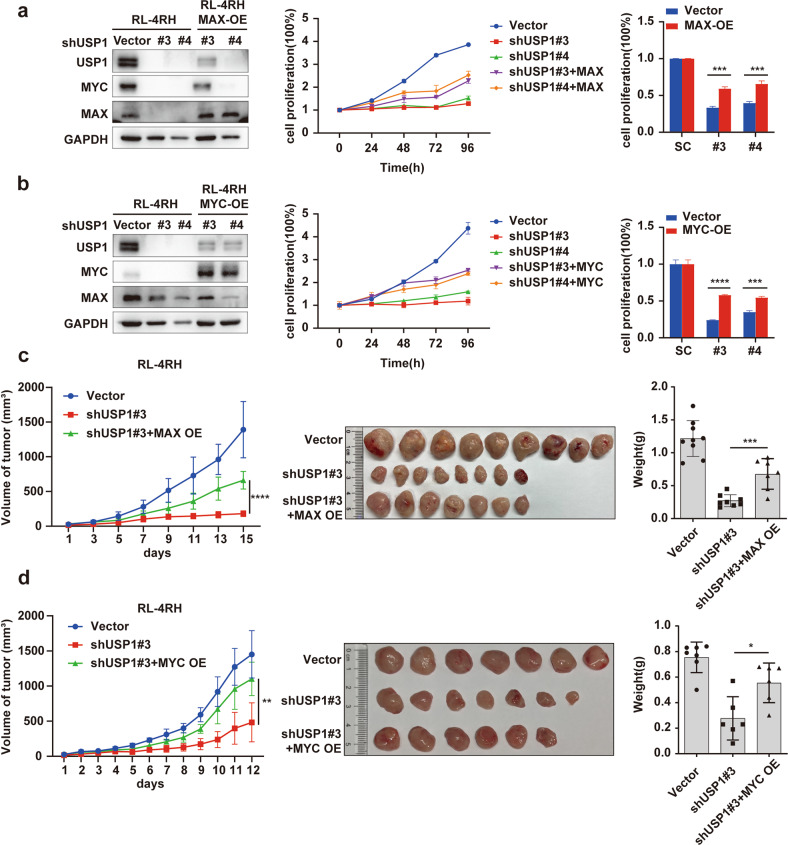
Overexpression of MAX or MYC rescued the inhibition of cell proliferation induced by USP1 knockdown in cell and mouse models of rituximab/chemotherapy resistant DLBCL. **a, b** RL-4RH cells overexpressing MAX or MYC were transduced with shRNA against USP1. The protein levels of USP1, MAX and MYC were measured by using western blotting assay (left panel), and the changes of cell proliferation were examined by using MTT assay (middle panel). The quantitative statistical results were shown 96 h after transduction (right panel). **c** The inhibition of cell proliferation induced by the USP1 knockdown was rescued by overexpression of MAX in the DLBCL xenotransplantation mouse model. **d** The inhibition of cell proliferation induced by USP1 knockdown was rescued by overexpression of MYC in the DLBCL xenotransplantation mouse model. Data are presented as mean ± SD from three independent experiments, *p < 0.05, **p < 0.01, ***p < 0.005, ****p < 0.001. Statistical analysis was performed with a paired *t* test.

To investigate whether USP1 knockdown-induced inhibition of cell proliferation was caused by the degradation of MAX or MYC protein in DLBCL cells, we constructed shRNAs against MAX or MYC and knocked down MAX or MYC in DLBCL cells. The results showed that knocking down MAX or MYC significantly inhibited cell proliferation (Supplementary Fig. S4d, e) and induced cell cycle arrest in DLBCL cells (Supplementary Fig. S4f, g). Subsequently, we overexpressed MAX or MYC and knocked down USP1 in RL-4RH cells. We found that overexpression of MAX or MYC significantly rescued USP1 knockdown-induced inhibition of cell proliferation in RL-4RH cells (Fig. 5a, b). Consistently, the decreased growth and weight of USP1-deficient tumors were significantly restored by MAX or MYC overexpression in the mouse model of rituximab/chemotherapy resistant DLBCL (Fig. 5c, d).

### USP1 inhibitor pimozide inhibited the proliferation of rituximab/chemotherapy resistant DLBCL cells in vitro and in vivo

**Fig. 6:**
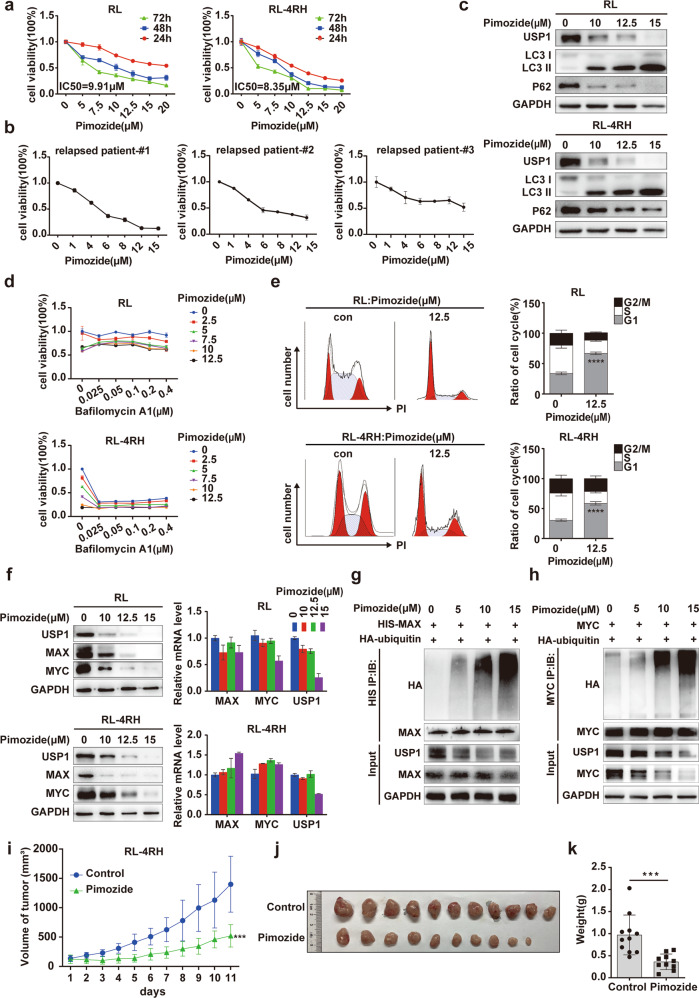
The USP1 inhibitor pimozide inhibited the proliferation of rituximab/chemotherapy resistant DLBCL cells in vitro and in vivo. **a** RL and RL-4RH cells were treated with pimozide at different concentrations for 24, 48 and 72 h, and the cell viability was measured by using MTT assay. **b** The primary lymphoma cells from a relapsed DLBCL patient were treated with pimozide at different concentrations for 24 h, and cell viability was measured by using MTT assay. **c** Autophagy-related proteins of RL and RL-4RH cells treated with pimozide were examined by using western blotting assay. **d** RL and RL-4RH cells were treated with the different combinations of pimozide and bafilomycin A1 for 72 hours and cell viability was examined by using MTT assay. **e** Changes of cell cycle in RL and RL-4RH cells treated with pimozide (12.5 μM) were examined by using flow cytometry assay. The representative picture of flow cytometry analysis (left panel), and the statistical results of flow cytometry experiments (right panel) were shown. **f** RL and RL-4RH cells were treated with pimozide (10 μM, 12.5 μM or 15 μM) for 48 hours, and the expression levels of USP1, MAX and MYC were examined by using western blotting (left panel) and quantitative real-time PCR (right panel) assays. **g**, **h** 293T cells overexpressing MAX or MYC were transfected with HA-ubiquitin and treated with pimozide at different concentrations for 24 h. MAX or MYC were immunoprecipitated with anti-HIS or anti-MYC antibody, and HA-ubiquitin was examined by using western blotting assay. **i**–**k** Pimozide treatment was performed in a DLBCL xenograft mouse model established with RL-4RH cells. Data are presented as mean ± SD from three independent experiments, *p < 0.05, **p < 0.01, ***p < 0.005, ****p < 0.001. Statistical analysis was performed with a paired *t* test.

Our study showed that USP1 knockdown delayed the growth of tumors in the RL-4RH cells-derived xenotransplantation mouse model of DLBCL, which suggested that USP1 is a potential therapeutic target for rituximab/chemotherapy resistant DLBCL. Pimozide, an USP1 inhibitor [22], has been approved by FDA for the treatment of psychiatric disorders, indicating that pimozide is readily available and safe [23]. Therefore, we investigated the function and mechanism of pimozide in DLBCL. We first detected the proliferation of DLBCL cell lines (RL, RL-4RH, SUDHL4, U2932, RIVA and TDM8 cells) treated with pimozide at different concentrations (5, 7.5, 10, 12.5, 15, and 20 μM) for 24, 48, or 72 h by using MTT assay. The results showed that pimozide inhibited the growth of these DLBCL cells in a time and concentration-dependent manner (Fig. 6a and Supplementary Fig. S5a). In addition, pimozide also significantly inhibited the proliferation of lymphoma cells from the relapsed and refractory DLBCL patients (Fig. 6b). Moreover, USP1 depletion greatly diminished the pimozide-mediated antiproliferation effects (Supplementary Fig. S5b). Next, the apoptosis of pimozide-treated DLBCL cells was examined by using flow cytometry analysis, and the results showed that pimozide treatment induced apoptosis in RL cells but not in RL-4RH cells (Supplementary Fig. S5c, d). Wright’s staining analysis also indicated that pimozide treatment induced apoptosis of RL cells (Supplementary Fig. S5e).

To investigate whether pimozide-induced cell death is dependent on autophagy, we evaluated the protein levels of p62 and LC3, the hallmark of autophagy activity, and found enhanced autophagic activity manifested by decreased p62 protein level and increased LC3 protein level in RL and RL-4RH cells with pimozide treatment (Fig. 6c). We treated these cells with the autophagy inhibitor, Bafilomycin A1, and the results showed that Bafilomycin A1 partially rescued cell death induced by pimozide (Fig. 6d). In addition, flow analysis showed that pimozide treatment induced cell cycle arrest at G0/G1 phase in RL and RL-4RH cells (Fig. 6e). To examine the changes in cell cycle-related proteins, we performed western blotting analysis in pimozide-treated RL and RL-4RH cells. The results showed that pimozide treatment upregulated the expression of cell cycle-arresting proteins such as P21 and P27, and downregulated proteins promoting cell cycle progression such as CyclinD3 and CDK2 (Supplementary Fig. S5f). Therefore, pimozide induced proliferation inhibition, apoptosis, autophagy and cell cycle arrest by targeting USP1 in DLBCL cells.

**Fig. 7:**
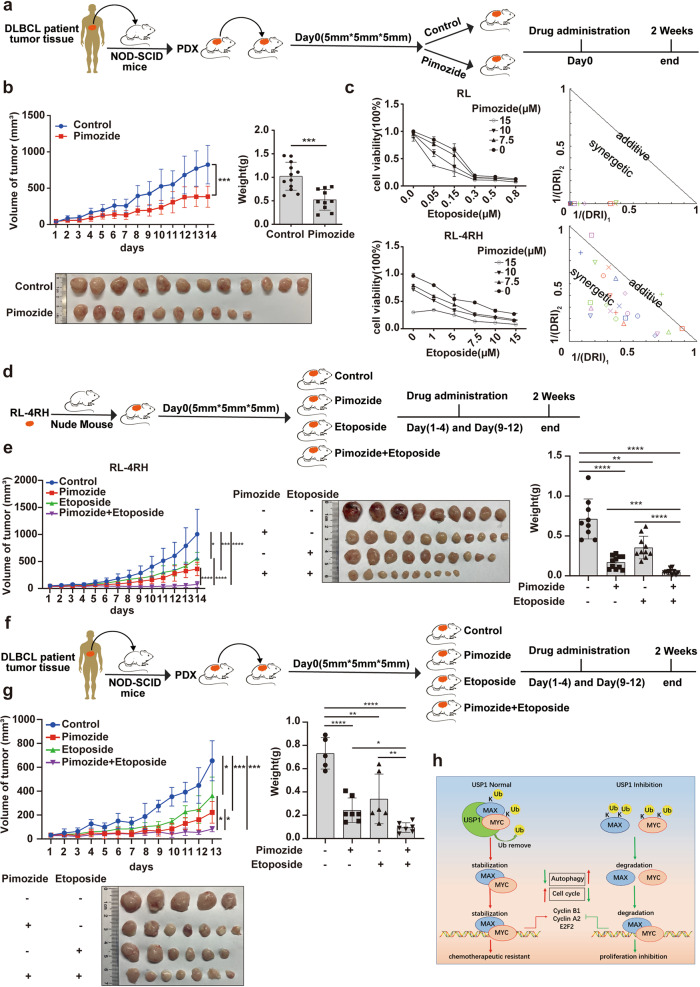
Pimozide treatment increased the sensitivity of rituximab/chemotherapy resistant DLBCL cells to etoposide. **a, b** Pimozide treatment was performed in a DLBCL xenograft mouse model established by using the DLBCL patient tumor tissues. **c** RL and RL-4RH cells were treated with the combination of pimozide and etoposide for 72 h. The viability of RL and RL-4RH cells was measured by using MTT assay, and the combinative effects of pimozide and etoposide on RL and RL-4RH cells were analyzed by using combosyn software. **d, e** Treatment with pimozide (15 mg/kg), etoposide (10 mg/kg) or the combination of pimozide (15 mg/kg) and etoposide (10 mg/kg) was performed in the DLBCL xenograft mouse model established by using RL-4RH cells. **f, g** Treatment with pimozide (15 mg/kg), etoposide (10 mg/kg) or the combination of pimozide (15 mg/kg) and etoposide (10 mg/kg) was performed in the PDX mouse model. **h** Schematic diagram of functions and molecular mechanisms of USP1 in DLBCL. Data are presented as mean ± SD, *p < 0.05, **p < 0.01, ***p < 0.005, ****p < 0.001. Statistical analysis was performed with a paired *t* test.

Since USP1 interacted with MAX/MYC and maintained their protein stability, we next examined whether pimozide could downregulate MAX/MYC and affect MYC target gene expression. We treated RL and RL-4RH cells with pimozide at the concentration of 10, 12.5 or 15 μM for 48 h, and found that pimozide treatment reduced the protein levels of MAX/MYC in RL and RL-4RH cells without affecting their mRNA levels (Fig. 6f). The expression of MYC target genes was examined by using qPCR assay, and we found that CyclinB1, CyclinA2 and E2F2 were significantly downregulated upon pimozide treatment (Supplementary Fig. S5g). To clarify how pimozide regulated MAX and MYC, we transfected 293T cells overexpressing MAX or MYC with HA-ubiquitin and treated these cells with pimozide for 24 h. The ubiquitination of these proteins was analyzed, and the results showed that pimozide treatment significantly increased the ubiquitination of MAX and MYC (Fig. 6g, h). To further investigate the effects of pimozide on rituximab/chemotherapy resistant DLBCL cells in vivo, we established a DLBCL xenograft mouse model with RL-4RH cells and treated the mice with pimozide. Compared with the control tumors, tumors with pimozide treatment displayed a reduced growth rate (Fig. 6i), and pimozide treatment significantly decreased the tumor weight (Fig. 6j, k). We also established a DLBCL PDX mouse model by xenotransplantation of NOD-SCID mice with primary tumor tissues of a DLBCL patient and treated these mice with pimozide. The results showed that pimozide treatment significantly delayed the progression of tumor in the DLBCL PDX mouse model (Fig. 7a, b) and decreased the expression levels of cell proliferation-related protein Ki67 in tumors (Supplementary Fig. S5h, i). In a conclusion, pimozide induced the degradation of MAX and MYC by upregulating their ubiquitination levels, thereby affecting the expression of MYC target genes, and ultimately inhibited the growth of DLBCL cells in vitro and in vivo.

### Pimozide treatment increased the sensitivity of rituximab/chemotherapy resistant DLBCL cells to etoposide

At present, the clinical treatment of lymphoma is mainly based on chemotherapy drugs. To explore whether pimozide had synergistic effect with chemotherapy drugs, we treated RL and RL-4RH cells with different concentrations of pimozide and chemotherapy drugs (dox, etoposide, hcl-gemcitabine or cisplatin) for 72 hours. The cell viability was detected by using the MTT assay, and the synergism of the drug combination was analyzed by using the ComboSYN software. The results showed that the combination of pimozide and etoposide but not dox, hcl-gemcitabine or cisplatin had synergistic effects on RL and RL-4RH cells (Fig. 7c). To further investigate the synergistic effects of pimozide and etoposide in vivo, we established the rituximab/chemotherapy resistant DLBCL xenograft mouse model with RL-4RH cells and treated the mice with pimozide and etoposide (Fig. 7d). Compared with the single drug group, we found that tumor growth was significantly inhibited upon the treatment in the combination group. The tumor weight of the combination group was significantly less than that of the single drug group (Fig. 7e). We also established a DLBCL PDX mouse model by xenotransplantation of NOD-SCID mice with primary tumor tissues of a DLBCL patient and treated these mice with pimozide and etoposide. The results showed that tumor growth was significantly inhibited in the combination group compared with the single drug group, and the tumor weight of the combination group was significantly less than that of the single drug group (Fig. 7f, g). These results indicated that pimozide may be used in combination with etoposide for the treatment of rituximab/chemotherapy resistant DLBCL (Fig. 7h). We also evaluated the therapeutic effects of the proteasome inhibitors and pimozide. We treated RL and RL-4RH cells with different concentrations of pimozide and proteasome inhibitor (MG132). The results showed that the IC50 of MG132 was significantly greater than pimozide in RL and RL-4RH cells (Supplementary Fig. S6a). Bruton Tyrosine Kinase (BTK) inhibitors have promising therapeutic effect on relapsed/refractory DLBCL [24–26]. To explore whether pimozide had synergistic effect with BTK inhibitors, we treated RL and RL-4RH cells with different concentrations of pimozide and BTK inhibitors (orelabrutinib, zanubrutinib). The results showed that the combination of pimozide and zanubrutinib but not orelabrutinib had synergistic effects on RL cells 72 h after the treatment (Supplementary Fig. S6b–i). To further investigate the synergistic effects of pimozide and zanubrutinib in vivo, we established the rituximab/chemotherapy resistant DLBCL xenograft mouse model with RL-4RH cells and treated the mice with pimozide (15 mg/kg) and zanubrutinib (2.5 mg/kg). The results showed that the combination of pimozide and zanubrutinib had no synergistic effects in the DLBCL xenograft mouse model (Supplementary Fig. S6j–l). In addition to this, to investigate the synergistic toxicity of pimozide and BTK inhibitor in vivo, we treated the C57BL/6 J mice with pimozide (15 mg/kg) and ibrutinib (5 mg/kg). The results showed that the combination of pimozide and ibrutinib had no significant cardio and coagulation side effect and pimozide did not deepen further toxicity (Supplementary Fig. S7a, b).

## Discussion

USP1, a member of the deubiquitylation enzyme family, has been reported to be involved in several malignancies, such as breast cancer, B/T cell acute lymphoblastic leukemia and glioblastoma [27–30]. In this study, we demonstrated the aberrant expression of USP1 in DLBCL patient specimens and cell lines. USP1 was highly expressed and associated with unfavorable prognosis in DLBCL. Both inhibition of USP1 with shRNA knockdown and pimozide treatment inhibited cell proliferation and induced autophagy and cell cycle arrest in DLBCL cells.

As a deubiquitylation enzyme, USP1 binds to the target proteins and maintains their stability by removing the ubiquitylation chain. It has been reported that USP1 can bind to ID proteins [11, 31], RPS16 [32], KDM4A/SIX1 [33, 34] and KPNA2 [30] in osteosarcoma, gastric cancer, hepatocellular carcinoma, prostate cancer and breast cancer respectively and maintain the stability of these proteins, which suggests that the target of USP1 could be cancer type-specific. Nevertheless, the target of USP1 in hematological malignancies including lymphoma, was not determined. In this study we have found a positive correlation between USP1 and MAX/MYC protein and identified MAX/MYC as the direct target of USP1 in rituximab/chemotherapy resistant DLBCL cells. Overexpression of USP1 prolonged the half-life of MAX/MYC protein and decreased the ubiquitination of MAX/MYC protein. MAX, like USP1, was also highly expressed in DLBCL cells compared with normal B cells, and DLBCL patients with high MAX expression had shorter OS. MAX is a member of basic helix-loop-helix leucine zipper (bHLHZ) family and forms homodimers and heterodimers with other family members, such as MAD, MXI1 and MYC [35]. As a transcription factor, MYC regulates the expression of a variety of genes involved in cell metabolism, proliferation, apoptosis and differentiation [36–39], and plays important carcinogenic roles in lymphoma [40, 41]. In MAX knockout Eµ-*Myc* mice, the levels of MYC protein and its direct target genes were significantly downregulated, and Eµ-*Myc*-induced lymphomas were almost completely inhibited, implying that MAX-MYC interactions are cell-background specific [19]. Our study showed that inhibition of USP1 by shRNA or pimozide treatment significantly increased the ubiquitination of MAX/MYC and reduced the protein levels of MAX/MYC in rituximab/chemotherapy resistant DLBCL cells, which led to the decrease of MYC target genes and the growth inhibition of lymphoma cells in the cell or patient-derived DLBCL mouse model. Thus, targeting USP1 decreased the levels of MAX/MYC and their target genes, and is a potential treatment for rituximab/chemotherapy resistant DLBCL.

Currently, R-CHOP is the standard treatment for DLBCL, but some patients have drug resistance during treatment, which has become difficult in the clinical treatment of lymphoma. MYC gene rearrangement rate in diffuse large B-cell lymphoma is about 10%, and R-CHOP treatment has poor efficacy for DLBCL patients with MYC gene rearrangement [42]. We found that the USP1 specific inhibitor pimozide affected the stability of MAX and MYC and inhibited the expression of MYC target genes in DLBCL cells. Pimozide can inhibit the proliferation of primary DLBCL cells and DLBCL cell lines and the growth of RL-4RH-derived xenograft tumors. What’s more, pimozide has been approved by FDA for the treatment of tourette’s syndrome, schizophrenia and chronic psychosis [43, 44], indicating that pimozide has low toxicity. Pimozide treatment sensitized the resistant DLBCL cells to etoposide, and the combination of pimozide with etoposide blocked the growth of the tumor in a mouse rituximab/chemotherapy resistant DLBCL model. Thus, pimozide combined with etoposide may represent an attractive strategy in treating patients with rituximab/chemotherapy resistant DLBCL. Overall, targeting USP1 by using pimozide is a potentially safe and effective treatment for R-CHOP resistant DLBCL.

## Supplementary information

Supplementary materials

Figure S1

Figure S2

Figure S3

Figure S4

Figure S5

Figure S6

Figure S7

## Data Availability

The datasets used and/or analyzed in the current study are available from the corresponding author on reasonable request.
