# Supplementary material for: Inhibition of USP1 reverses the chemotherapy resistance through destabilization of MAX in the relapsed/refractory B-cell lymphoma
Source: Leukemia. 2022 Nov 9;37(1):164–77. doi: 10.1038/s41375-022-01747-2 (PMC9883169; doi:10.1038/s41375-022-01747-2)
Supplement: Supplementary file 1 — Supplementary materials [file 41375_2022_1747_MOESM1_ESM.docx]

**Supplemental Materials and Methods**

**Reagents and antibodies**

The chemicals used in the experiments: protease inhibitor cocktail (Biomake, B14002), PMSF (MCE, HY-B0496), MG132 [Sigma/flu/Ald, (R)-MG132], Cycloheximide (CST, 2112S), pimozide (Sigma/flu/Ald, P1793), etoposide (HENGRUI-pharma), MTT (Amresco), propidium iodide (PI) (Sigma, P4170), Matrigel (BD biocoat, 354234), APC-Annexin V (BD Pharmingen, 550474) and Duolink® In Situ Detection Reagents Orange (Sigma, DUO92007), Zanubrutinib (MCE, HY-101474A), Ibrutinib (MCE, HY-10997), Bafilomycin A1 (MCE, HY-100558). The antibodies used in our experiments: anti-USP1 (Proteintech, 14346-1-AP), GAPDH (Proteintech, 354358), CyclinA2 (CST, 4656), CyclinB1 (CST, 12231T), MAX (CST, 4739S), c-MYC (Proteintech, 10828-1-AP), HA (CST, 3724S) or FLAG (Sigma/flu/Ald, F7425) antibodies, anti-FLAG(R) M2 magnetic beads affinity isolated antibody (Sigma/flu/Ald, M8823) and cell cycle regulation antibody sampler kit (CST, 9932T).

**Isobolographic analysis**

Determination of the synergistic of etoposide and pimozide was performed by using the combination index (CI) method described by Chou and Talalay (CalcuSyn software, Biosoft). When at least 80% of CI values for a combination were less than one, the drug combination was synergistic.

**Lentivirus production and infection**

293T cells were transfected with gene plasmids (shSC, shUSP1-#3 or shUSP1-#4) and packaging plasmids (pspax2 and pMD2G) by calcium phosphate-DNA precipitation. Virus supernatants were collected 48 or72 hours after transfection and filtered with 0.45 μM sterile millex filters. 5 x 10^5^ cells were infected with 1 mL fresh filtered virus supernatant and 10 μg/mL of Polybrene (Sigma), and then centrifuged at 1600 rpm for 90 minutes at room temperature. The medium was changed 8 to 10 hours after the virus infection. Stably transfected cells were selected with puromycin (1 μg/mL). The cells were collected and used for subsequent analysis 96 hours after transfection.

**Lentivirus-mediated gene knockdown**

The control shRNA was synthesized with a scrambled sequence. The shRNAs were constructed with shRNA-sequences from the GPP Web Portal. Virus preparation and cell infection were performed as described above. The efficiency of knockdown was measured 4 days after infection by using western blotting and q-PCR analysis. The sequences for shRNAs against USP1, MAX or MYC were shown in Table S1.

**RT-PCR and RT-qPCR**

The total RNA was extracted with TRIzol Reagent (Invitrogen) and cDNA was synthesized by PrimeScript™ RT reagent Kit with gDNA Eraser (TaKaRa #RR047A). SYBR® Premix Ex Taq™ GC (ROCHE) was used for quantitative real-time-PCR (RT-qPCR) analysis, and Glyceraldehyde-3-phosphate dehydrogenase (GAPDH) was used as an internal control of RNA integrity. RT-qPCR was performed in triplicate. The sequences of primers used were given in Table S2.

**Flow cytometry**

All the flow cytometry experiments were performed on the Beckman Coulter cell analyzer and analyzed with FlowJo V10 or MFLT32 for apoptosis and cell cycle. For apoptosis analysis, 1 × 10^5^ cells were incubated with APC-Annexin V and propidium iodide (PI, 10 µg/mL) at room temperature for 15 minutes in 1 × binding buffer, and then analyzed on a Beckmann Gallios cell analyzer machine. For cell cycle analysis, 1 × 10^6^ cells were fixed with 70% alcohol over 24 hours at -20 °C, and then disposed with RNase (100 µg/mL) for 30 minutes at 37 °C. The cells were washed once with PBS and incubated with PI (10 µg/mL) for 15 minutes.

**Mouse model and drug administration**

5 x 10^6^ RL-4RH cells were suspended in RPMI medium and mixed with 50 µL matrigel to 100 µL. The cells were injected subcutaneously into both flanks of female nude mice (5- or 6-week-old). Treatment started when the tumor became about 5 mm × 5 mm in surface (day 0). The lymphoma PDX mouse model was a kind gift given from Dr. Li Wang (Ruijin Hospital affiliated to Shanghai Jiao Tong University School of Medicine). Tumor volumes were calculated as 0.5 × a × b^2^ (‘a’ is the length and ‘b’ is the width). The approval number of committees for ethical review is SINH-2021-WL-3.

**Immunohistochemistry**

Formalin-fixed, paraffin-embedded tissue slides were deparafﬁnized and rehydrated. These samples then had antigen retrieval at 97 °C in Tris/EDTA (50 mM Tris, 2 mM EDTA, PH9.0) for 15 minutes and cooled naturally to room temperature. Next, the slides were immersed in 3% hydrogen peroxide for 15 minutes and blocked by 1% bovine serum albumin (BSA) for 20 minutes at room temperature. The slides were incubated with primary antibody against USP1 (Proteintech) overnight at 4 °C at a dilution of 1:1000 and secondary antibody for 1 hour at room temperature. Between each incubation step, the slides were washed three times with PBS (pH 7.4), and then the slides were visualized using the Dako Envision System (Dako, Glostrup, Denmark). Finally, the slides were counterstained with hematoxylin, dehydrated with ethanol and mounted with coverslips. The slides without incubation with primary antibodies served as negative controls.

**Proximity ligation assay (PLA)**

3 x 10^4^ cells were plated on a chamber slide and fixed with 4% paraformaldehyde for 30 minutes at RT (room temperature). Permeabilization was obtained by using 0.5% Triton X-100 in PBS. The cells were incubated with 5% BSA in PBS for 60 minutes at RT and primary antibodies overnight at 4 °C. The two oligonucleotide-linked PLA probes were mixed and diluted (1:5 or 1:10) in the antibody diluent. The cells were incubated with the solution for 1 hour at 37 °C and washed with wash buffer twice. Ligation: the cells were incubated with the ligase solution [1:40 dilution in ligation buffer (5x) with Milli-Q water], for 30 minutes at 37 °C, and washed with wash buffer A twice. Amplification: the cells were incubated with the premixed polymerase and nucleotide solution[1:80 using amplification red(5x) in Milli-Q water and mix] for 120 minutes at 37 °C. Gently wash the slides twice with 1x wash buffer B , and dry the slides at RT. Slides were sealed using a tablet sealer containing DAPI. The images were analyzed using fluorescence microscopy.

**Enzyme linked immunosorbent assay**

Peripheral blood of mice was diluted with EDTA and centrifuged at 3000rpm for 20 minutes at 4 °C, and the supernatant was obtained. The sample to be tested first needs to be diluted with sample dilution, and then add 50 µL of the sample to be tested to the enzyme-labeled coated plate. Incubate the plate at 37 °C for 30 minutes, then add the washing liquid to clean 5 times. Add 50 µL HRP-conjugated reagent, sealing the plate and incubated at 37 °C for 30 minutes, remove the sealing membrane, discard the liquid, add the washing liquid to clean 5 times. Add the chromogenic agent A 50 µL, then add the chromogenic agent B 50 µL, and incubate at 37 °C for 10 minutes in the dark. Add 50 µL of stop solution and measure the absorbance at 450nm wavelength.

**Statistical analysis and bioinformatics**

The clinical data analysis was performed using SPSS 23.1 (SPSS Inc, Chicago). Kaplan-Meier survival curves were constructed for survival analyses, and differences were tested by the log-rank test. The data of living patients at the end of the study were censored. The correlation between the USP1 expression and clinical variables was tested by the Pearson Chi Square test. All P values were two-sided, and the results were considered significant if P < 0.05. Determination of the synergistic of etoposide and pimozide was performed by using the combination index (CI) method described by Chou and Talalay (CalcuSyn software, Biosoft). It was synergistic when at least 80% of CI values for a combination were less than one. All other statistical analyses were performed using the GraphPad Prism software. Unpaired student’s t test was used for comparison of groups of samples of equal variance. The IC50 was calculated using GraphPad Prism software.

**Supplementary Figure legend**

**Figure S1. USP1 is highly expressed in DLBCL and associated with poor prognosis. a** Multivariate analysis of USP1 associated with PFS and OS. **b** The expression of USP1 in different DLBCL subtypes was analyzed using immunohistochemistry analysis.

**Figure S2. Knockdown of USP1 inhibited DLBCL cell proliferation and induced apoptosis and cell cycle arrest. a, b** The expression of USP1 was examined by using western blotting and quantitative real-time PCR assays, and the cell proliferation was detected by using MTT assay in RL and RL-4RH cells with USP1 knockdown. **c** Wild-type USP1 and catalytically inactive mutant of USP1 were overexpressed in RL-4RH cells with USP1 knockdown. **d** Apoptosis of RL and RL-4RH cells with USP1 knockdown was measured by using flow cytometry assay. Representative dot plots of flow cytometry analysis (left panel) and the statistical results of flow cytometry experiments (right panel) were shown. **e** Observation of the apoptotic bodies by using Wright’s staining analysis. **f** Changes in the cell cycle were examined by using flow cytometry assay in U2932 and SUDHL4 cells with USP1 knockdown. The representative picture of flow cytometry analysis (left panel), and the statistical results of flow cytometry experiments (right panel) were shown. Data are presented as mean ± SD from three independent experiments, *p < 0.05, **p < 0.01, ***p < 0.005, ****p < 0.001. Statistical analysis was performed with a paired t test.

**Figure S3. USP1 interacted with MAX and MYC in rituximab/chemotherapy resistant DLBCL cells. a** Analysis of a GEO dataset showed that expression levels of MAX in DLBCL cells were significantly higher than that in normal B cells. **b** Multivariate analysis of the MAX correlation for PFS and OS. **c, d** Immunohistochemical analysis was performed to analyze the expression of MAX in different DLBCL subtypes. **e, f** GST-USP1, HIS-MAX and HIS-MYC prokaryotic expression plasmids were constructed and the GST-pull down assays were performed. **g** Immunohistochemical staining analysis for USP1 and MAX in primary DLBCL samples. **h** Correlation analysis of USP1 and MAX in primary DLBCL samples. Data are presented as mean ± SD from three independent experiments, *p < 0.05, **p < 0.01, ***p < 0.005, ****p < 0.001. Statistical analysis was performed with a paired t test.

**Figure S4. Knockdown of MAX or MYC inhibited the proliferation of rituximab/chemotherapy resistant DLBCL cells and induced cell cycle arrest.** **a** USP1 was knocked down in RL-4RH cells and the expression of the MYC target genes was examined by using qPCR assay. **b** The K57R mutation decreased the level of MAX ubiquitylation in 293T cells. **c** USP1 knockdown enhanced ubiquitylation of MAX^WT^ but not MAX^K57R^ mutant. **d** The expression of MAX was examined by using western blotting assay in RL-4RH cells transduced with shRNA against MAX (left panel). The proliferation of RL, RL-4RH and SUDHL4 cells transduced with MAX was examined by using MTT assay (right panel). **e** The expression of MYC was examined by using western blotting assay in RL-4RH cells transduced with shRNA against MYC (left panel). The proliferation of RL and RL-4RH cells with MYC knockdown was measured by using MTT assay (right panel). **f, g** Changes in the cell cycle were measured by using flow cytometry assay. The representative pictures of flow cytometry analysis (left panel) and the statistics results of each phase (right panel) were shown. Data are presented as mean ± SD from three independent experiments, *p < 0.05, **p < 0.01, ***p < 0.005, ****p < 0.001. Statistical analysis was performed with a paired t test.

**Figure S5. Pimozide inhibited DLBCL cell proliferation and induced cell cycle arrest. a** DLBCL cells were treated with pimozide at different concentrations for 24, 48 or 72 hours, and the cell viability was measured by using the MTT assay. **b** Proliferation of the RL-4RH cells with USP1 knockdown (shUSP1) or control cells, treated with DMSO or pimozide (7.5 or 12.5 μM) for 48 hours. **c, d** Apoptosis of RL and RL-4RH cells treated with pimozide at different concentrations was measured by using flow cytometry assay. **e** RL cells were treated with pimozide (12.5 μM) and the apoptotic bodies were observed by using Wright’s staining assay. **f** RL and RL-4RH cells were treated with 12.5 μM pimozide for 24, 48, 72 hours, and the cell cycle-related proteins were measured by using western blotting assay. **g** RL and RL-4RH cells were treated with pimozide (10 μM, 12.5 μM or 15 μM) for 48 hours, and the MYC target genes were examined by using qPCR assay. **h, i** The expression levels of Ki67 in RL-4RH cells or PDX xenograft tumor treated with pimozide or the vehicle control were determined by using IHC assay. Data are presented as mean ± SD from three independent experiments, *p < 0.05, **p < 0.01, ***p < 0.005, ****p < 0.001. Statistical analysis was performed with a paired t test.

**Figure S6. Pimozide has no synergistic effects with** **proteasome inhibitor or Bruton Tyrosine Kinase (BTK) inhibitor *in* *vitro* and *in vivo*. a** RL and RL-4RH cells were treated with different concentrations of pimozide and proteasome inhibitor (MG132) for 24 hours, 48 hours and 72 hours. The MTT assay was performed to detect cell viability. **b-i** RL and RL-4RH cells were treated with different concentrations of pimozide and BTK inhibitor (orelabrutinib, zanubrutinib) for 48 hours and 72 hours. The the MTT assay was performed to detect cell viability and the synergism of the drug combination was analyzed by using the ComboSYN software. **j-l** Treatment with pimozide (15 mg/kg), zanubrutinib (2.5 mg/kg) or the combination of pimozide (15 mg/kg) and zanubrutinib (2.5 mg/kg) was performed in the DLBCL xenograft mouse model established by using RL-4RH cells. Data are presented as mean ± SD from three independent experiments, *p < 0.05, **p < 0.01, ***p < 0.005, ****p < 0.001. Statistical analysis was performed using paired t-test.

**Figure S7. Effect of pimozide and ibrutinib on cardiovascular and coagulation *in vivo*.** **a** HE and Masson staining of heart slices. **b** C57BL/6J mice were treated with pimozide (15 mg/kg), ibrutinib (5 mg/kg) or the combination of pimozide (15 mg/kg) and ibrutinib (5 mg/kg). Myoglobin (MYO), troponin (Tn), creatine kinase isoenzyme (CK-MB) and prothrombin (PT) were detected. Data are presented as mean ± SD from three independent experiments, *p < 0.05, **p < 0.01, ***p < 0.005, ****p < 0.001. Statistical analysis was performed using paired t-test.

**Supplementary Tables**

**Table S1. Sequences for shRNAs against USP1, MAX or MYC.**

| **Gene** | **Primer sequence** |
| --- | --- |
| **shUSP1#3**  **shUSP1#4**  **shMAX#1**  **shMAX#2**  **shMYC#1**  **shMYC#10** | 5’-GCTAGTGGTTTGGAGTTTGAT-3’  5’-CCAGTGACCAAACAGGCATTA-3’  5’-CTGAGTGAATTGTACCTATTT-3’  5’-ACACACACCAGCAAGATATTG-3’  5’-CTGAGACAGATCAGCAACAA-3’  5’-GAACTATGACCTCGACTACGA-3’ |

**Table S2. Primer sequences for qRT-PCR.**

| **Gene** | **Primer sequence** |
| --- | --- |
| **USP1** | F 5’-GCTCTAAAGGATGAAGCCAATCAA-3’ |
|  | R 5’-ACTAGCCTGGAGCTGTTCAACC-3’ |
| **MAX** | F 5’-TTGACGACCTCAAGCGGCAGAA-3’ |
|  | R 5’-TTGGCGTTGGTGTAGAGGCTGT-3’ |
| **MYC** | F 5’-CCTGGTGCTCCATGAGGAGAC-3’ |
|  | R 5’-CAGACTCTGACCTTTTGCCAGG-3’ |
| **GAPDH** | F 5'-GTCTCCTCTGACTTCAACAGCG-3' |
|  | R 5'-ACCACCCTGTTGCTGTAGCCAA-3' |
| **Cyclin B1** | F 5'-GACCTGTGTCAGGCTTTCTCTG-3'  R 5'-GGTATTTTGGTCTGACTGCTTGC-3' |
| **Cyclin A2** | F 5'-CTCTACACAGTCACGGGACAAAG-3'  R 5'-CTGTGGTGCTTTGAGGTAGGTC-3' |
| **E2F2** | R 5'-CTCTCTGAGCTTCAAGCACCTG-3'  R 5'-CTTGACGGCAATCACTGTCTGC-3' |

Abbreviations: F, forward primer; R, reverse primer
