# Supplementary figures and images for: Inhibition of USP1 reverses the chemotherapy resistance through destabilization of MAX in the relapsed/refractory B-cell lymphoma
Source: Leukemia. 2022 Nov 9;37(1):164–77. doi: 10.1038/s41375-022-01747-2 (PMC9883169; doi:10.1038/s41375-022-01747-2)

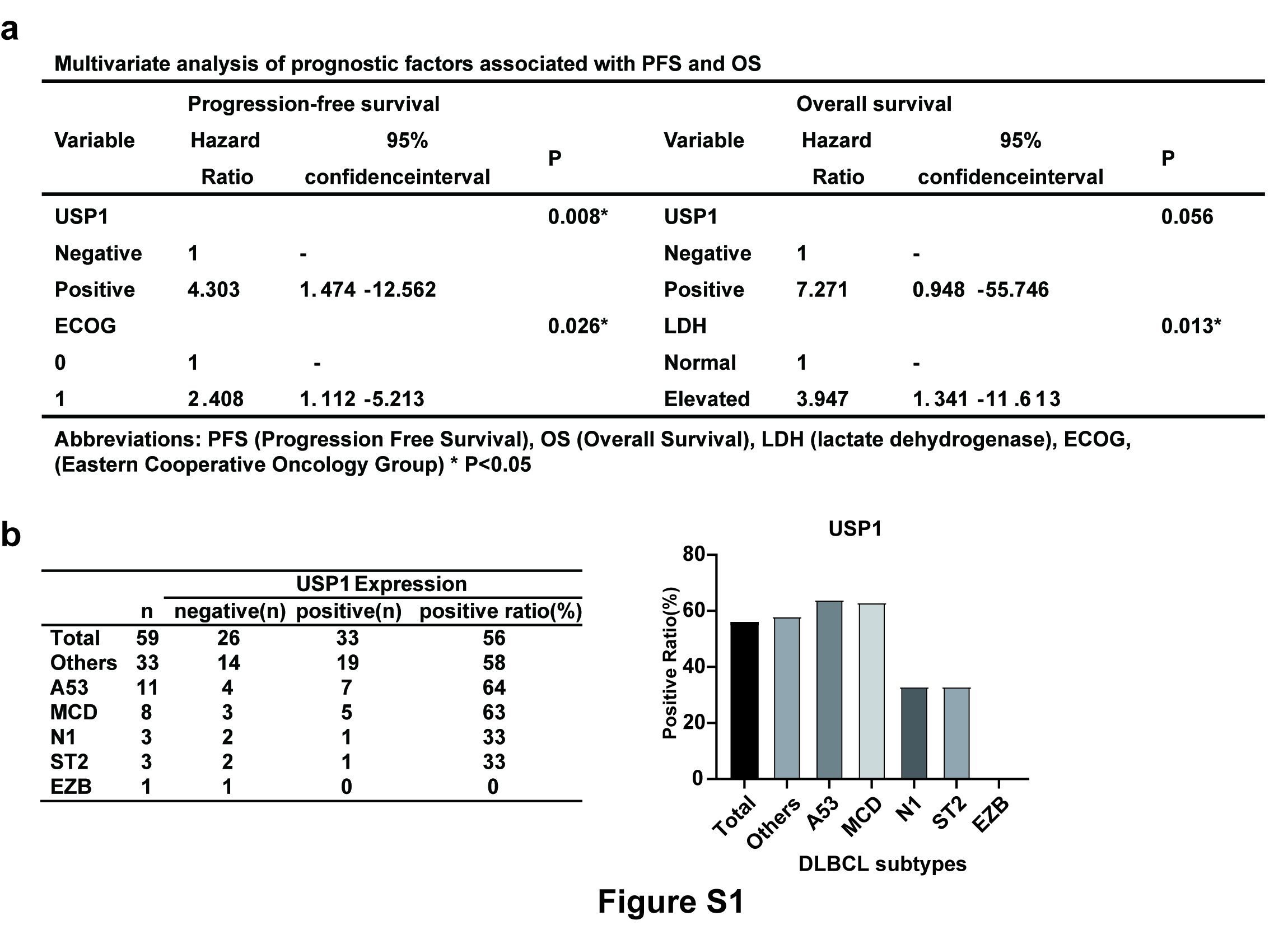

Supplement: Supplementary file 2 — Figure S1 [file 41375_2022_1747_MOESM2_ESM.jpg]

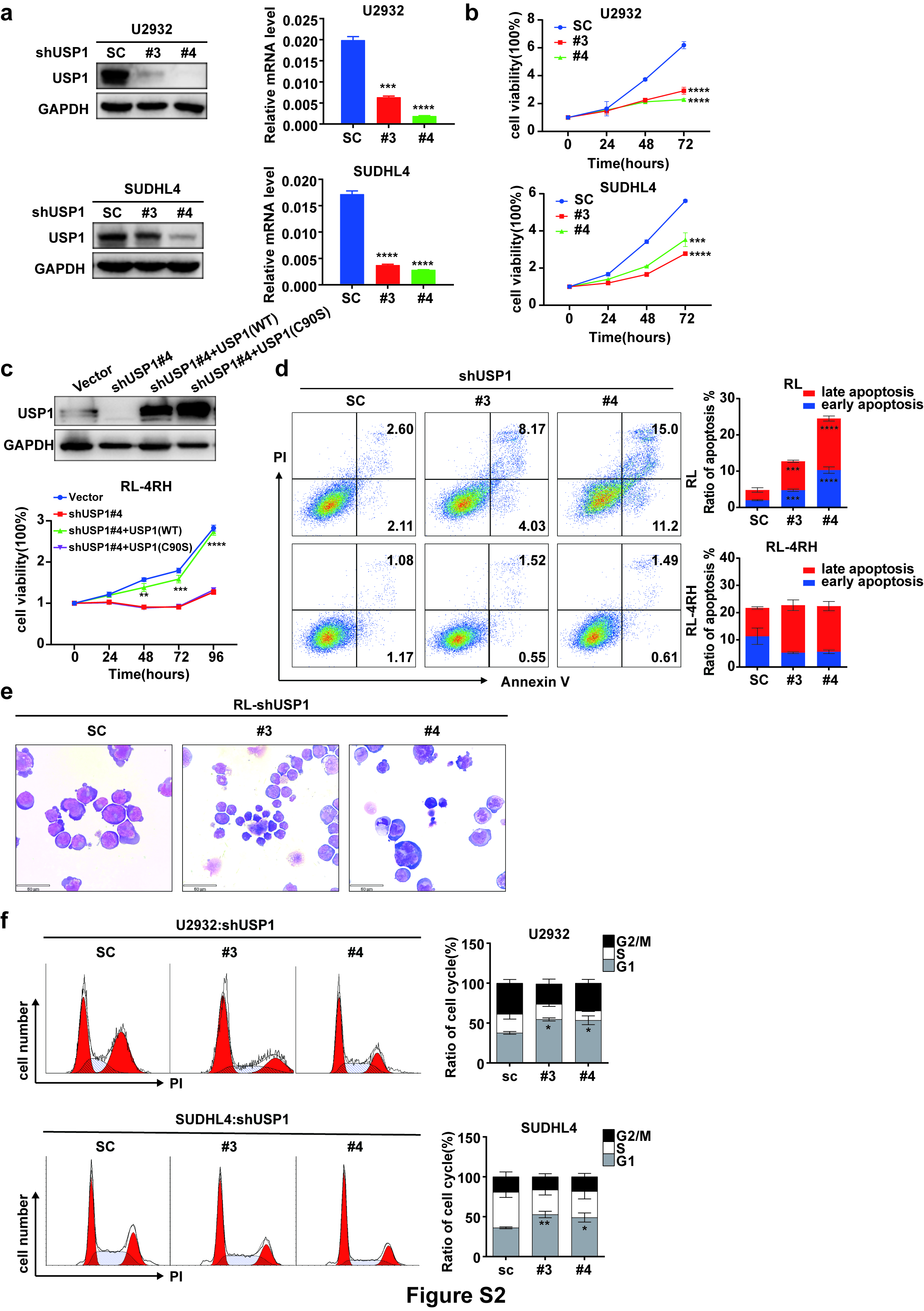

Supplement: Supplementary file 3 — Figure S2 [file 41375_2022_1747_MOESM3_ESM.jpg]

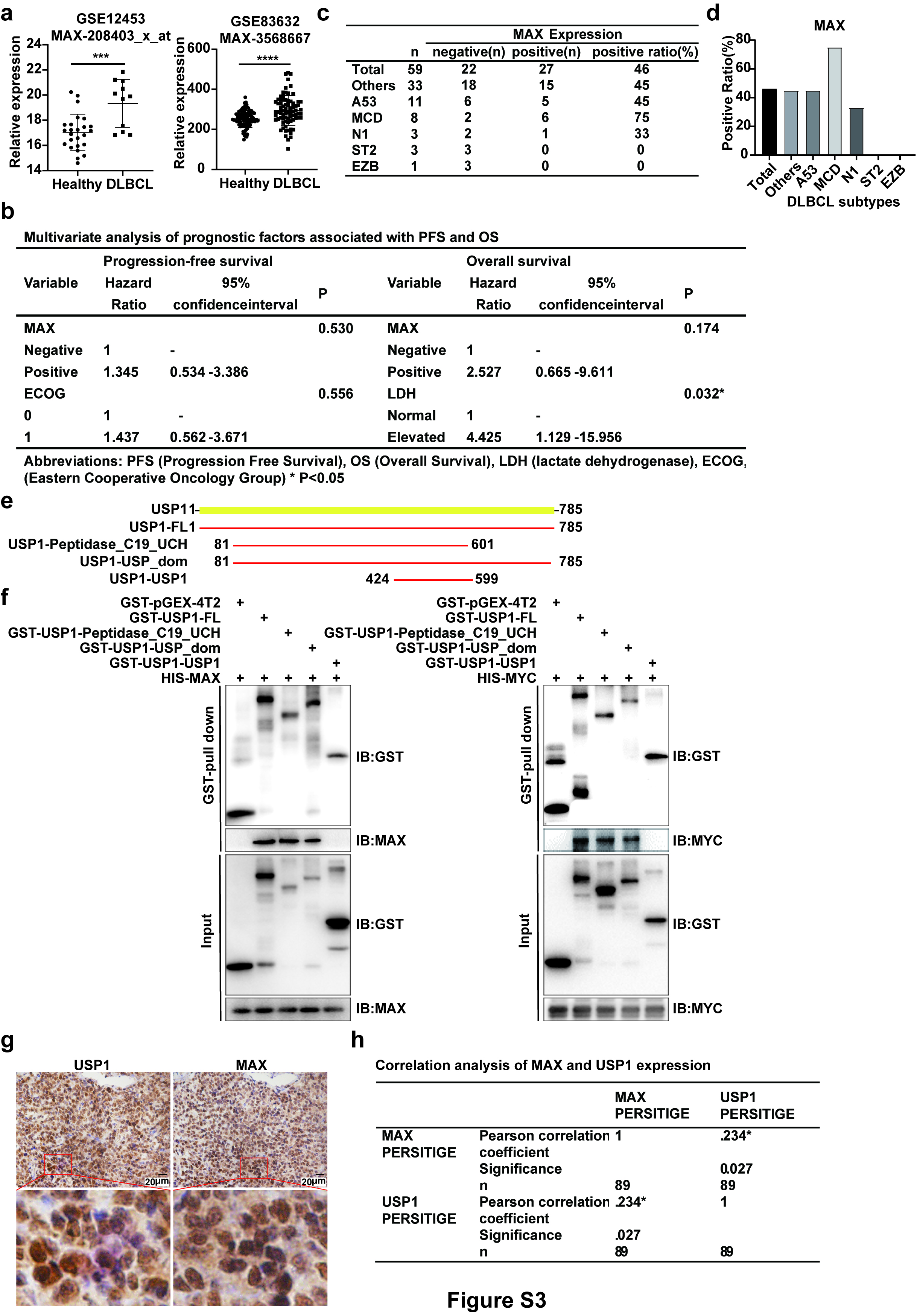

Supplement: Supplementary file 4 — Figure S3 [file 41375_2022_1747_MOESM4_ESM.jpg]

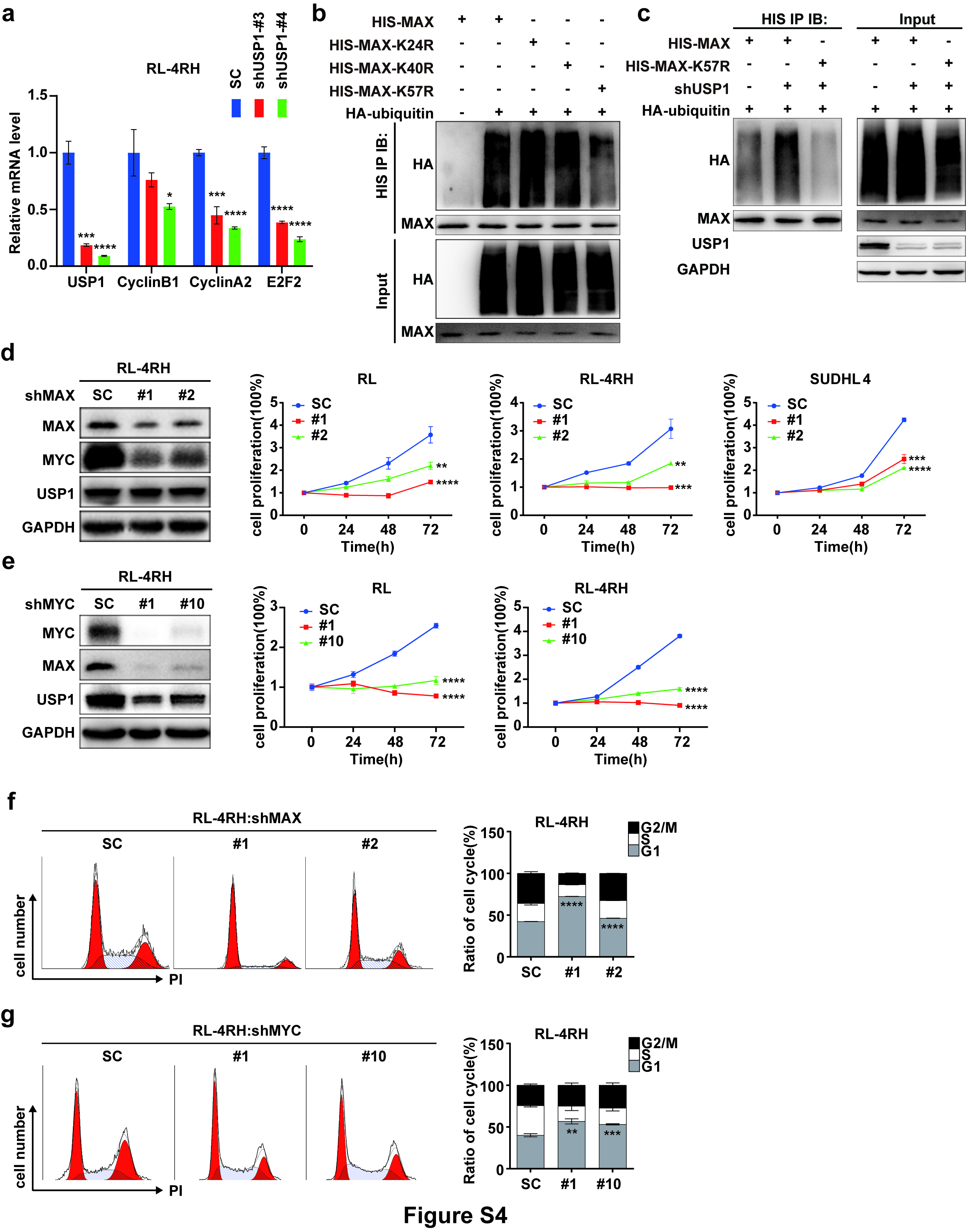

Supplement: Supplementary file 5 — Figure S4 [file 41375_2022_1747_MOESM5_ESM.jpg]

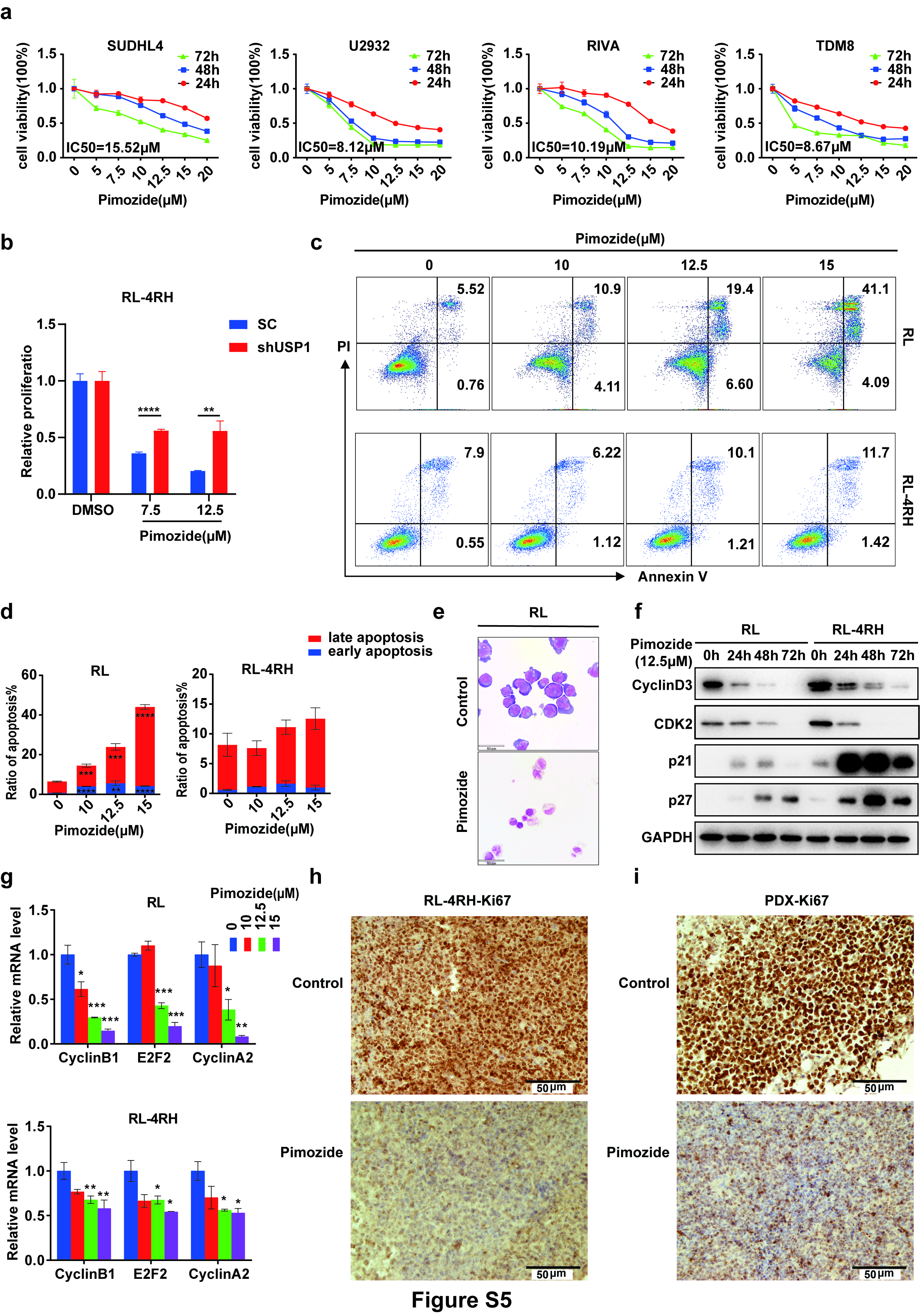

Supplement: Supplementary file 6 — Figure S5 [file 41375_2022_1747_MOESM6_ESM.jpg]

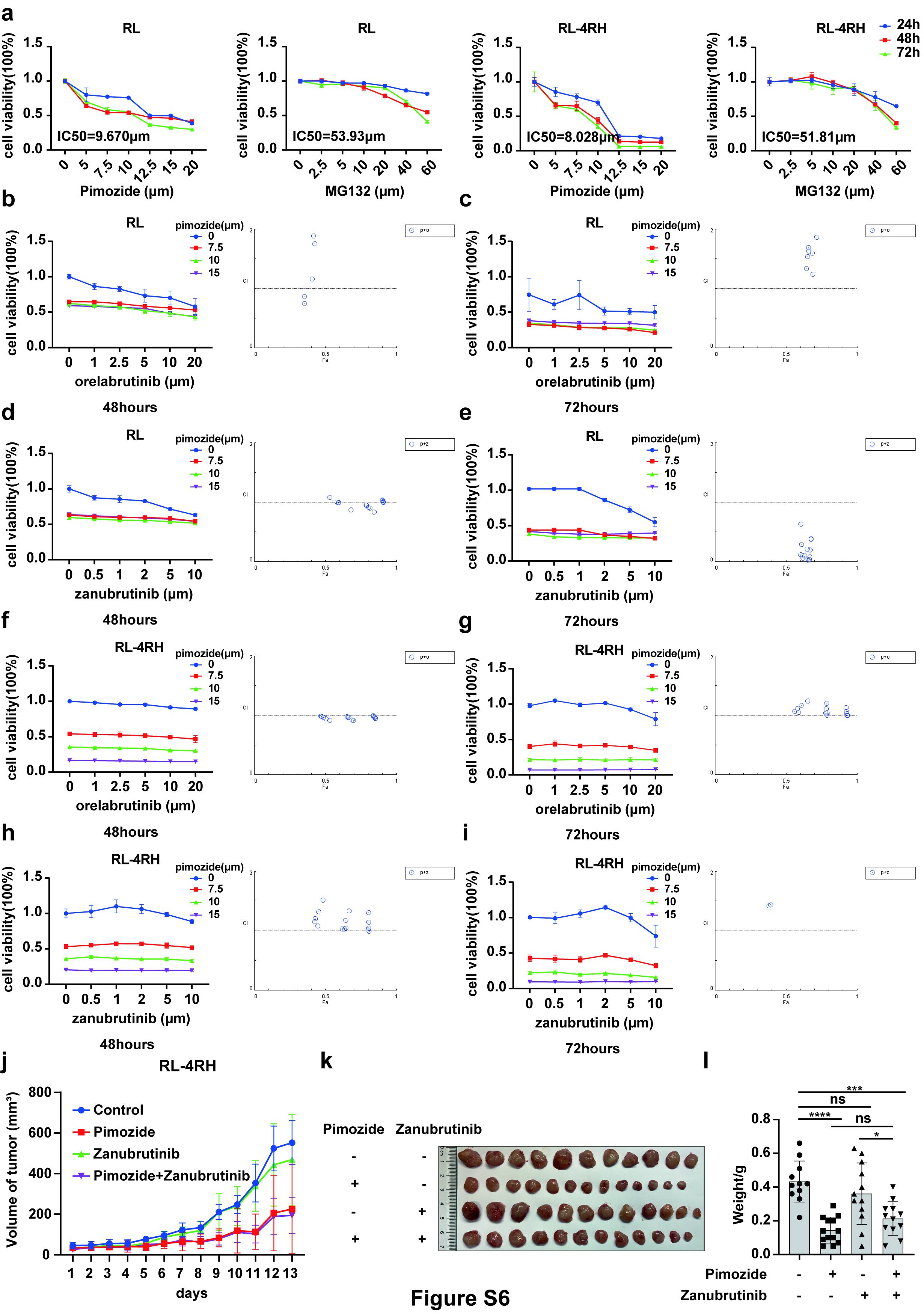

Supplement: Supplementary file 7 — Figure S6 [file 41375_2022_1747_MOESM7_ESM.jpg]

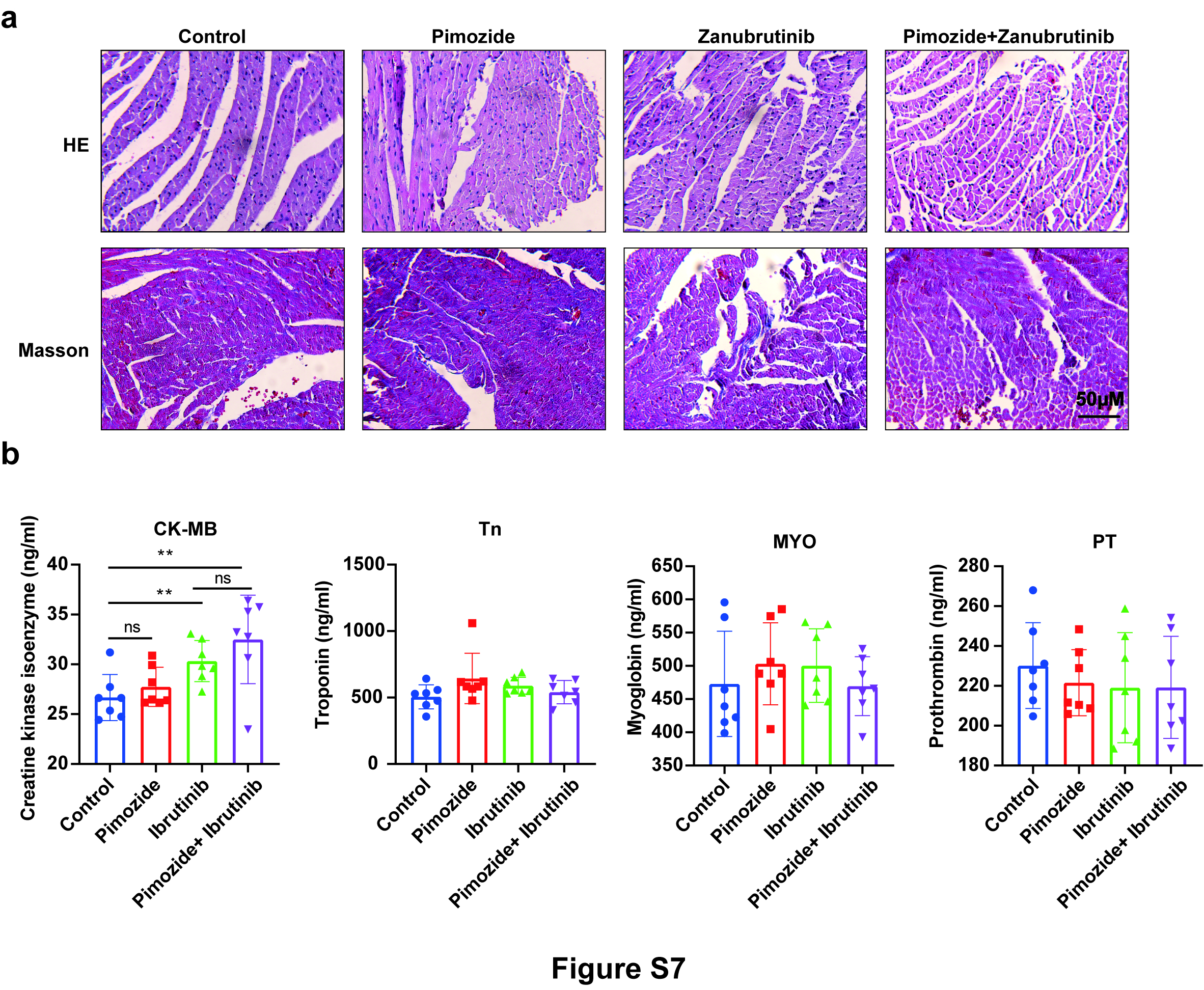

Supplement: Supplementary file 8 — Figure S7 [file 41375_2022_1747_MOESM8_ESM.jpg]
